# Microbial Primer: Biosurfactants – the ABCs of microbial surface-active metabolites

**DOI:** 10.1099/mic.0.001604

**Published:** 2025-09-05

**Authors:** Maude Dagenais Roy, Eric Déziel

**Affiliations:** 1 Institut National de la Recherche Scientifique (INRS) – Centre Armand-Frappier Santé Biotechnologie, Laval, Québec, Canada

**Keywords:** amphiphiles, microbial surfactants, secondary metabolites

## Abstract

Microbial surfactants (biosurfactants) are low-molecular-weight amphiphilic secondary metabolites synthesized by a wide range of micro-organisms, including bacteria, yeasts and fungi. These compounds reduce surface and interfacial tension, promote emulsification and self-assemble into supramolecular structures such as micelles. Their remarkable structural diversity reflects the metabolic complexity of their microbial producers. In this primer, we outline shared features across biosurfactant-producing organisms, focusing on biosynthetic pathways, biological functions and regulatory mechanisms. The study of biosurfactants lies at the intersection of ecological, biotechnological and medical research, offering valuable insights into microbial ecology and promising avenues for sustainable innovation.

## Microbial surfactants: low-molecular-weight amphiphilic metabolites

A variety of micro-organisms are capable of synthesizing and releasing low-molecular-weight amphiphilic molecules into their extracellular environment. These metabolites are often referred to as biosurfactants, which comprise a diverse array of compounds naturally produced by bacteria, yeasts and fungi. Biosurfactants possess an amphiphilic structure, featuring a hydrophobic portion, typically a fatty acid, and a hydrophilic portion, such as a sugar, peptide or amino acid.

Biosurfactants are specific types of molecules that belong to a broader category known as SURFace-ACTive AgeNTS or surfactants. They share amphiphilicity, enabling them to accumulate at interfaces and decrease their interfacial tension by reducing the free energy at these boundaries. An interface is defined as the boundary between two phases of different polarities, such as liquid-gas, liquid-liquid, liquid-solid, solid-solid and solid-gas. The free energy associated with these interfaces represents the work needed to expand the contact area between the two phases. Surfactants help to reduce this energy, making it easier for one phase to disperse into another.

One notable effect of lowering surface tension is the promotion and stabilization of emulsions. Emulsions are mixtures of two immiscible liquids, such as water and oil, where fine droplets of one phase are dispersed throughout the other. Wetting and dispersion are other properties of surfactants that involve their interaction with solid surfaces and liquids. Surfactants, by decreasing surface tension, can help liquids spread more easily on surfaces. Within a liquid, they stabilize solid particles in suspension and effectively prevent their aggregation. Additionally, certain types of surfactants contribute to foam formation by adsorbing at the air–water interface. This process not only traps gas bubbles but also enhances their stability in water.

Surfactants can form supramolecular structures, one of the most notable being micelles. Micelles are spherical formations that emerge when the concentration of the surfactant in a solution exceeds the critical micelle concentration. At lower concentrations, surfactant molecules tend to adsorb at interfaces; however, upon reaching this critical threshold, they spontaneously assemble into aggregates, such as micelles. This behaviour is due to the hydrophobic tail of the surfactant molecules, which tends to avoid water and thus cluster together. Micelles play a significant role in solubilizing hydrophobic molecules within an aqueous solution by capturing them in their hydrophobic centres. Additionally, surfactants can form bilayer structures known as vesicles, which trap hydrophilic molecules instead of hydrophobic ones.

In the literature, many molecules are mistakenly classified as biosurfactants. Herein, we define biosurfactants as low-molecular-weight amphiphiles produced by micro-organisms, typically as secondary metabolites. According to this definition, surface-active molecules derived from plant extracts are bio-based surfactants, not biosurfactants. Furthermore, some micro-organisms produce high-molecular-weight bioemulsifiers, such as emulsans, polymers and glycoproteins, which stabilize emulsions. Nevertheless, these are not considered surfactants per se, as they do not reduce surface tension and lack the amphiphilic nature characteristic of true biosurfactants.

## Diversity of microbial producers and chemical structures

The history of biosurfactants can be traced back to the early twentieth century, although their true significance was not fully appreciated until several decades later. The first documented observation of compounds produced by micro-organisms that could reduce surface tension was reported in 1946 when a glycolipid (GL) from *Pseudomonas aeruginosa* (then known as *Pseudomonas pyocyanea*) was described. In the years that followed, these compounds were identified as rhamnolipids (RLs) and characterized further. Some biosurfactants were initially recognized for their antimicrobial properties, with one notable example being polymyxins, produced by *Paenibacillus polymyxa* (formerly *Bacillus polymyxa*). Polymyxins were originally identified as antibiotics in 1947. However, they were later demonstrated to be cyclic lipopeptide (CLP) biosurfactants, and their antimicrobial activity was associated with their amphiphilic nature. In the ensuing decades, various classes of biosurfactants were identified, including sophorolipids from *Starmerella bombicola* (previously known as *Torulopsis bombicola*, then *Candida bombicola*) and surfactins synthesized by *Bacillus subtilis*. To date, over 2,000 distinct structures of biosurfactants have been identified, including different types of biosurfactants and their congeners. This remarkable diversity highlights the broad range of microbial producers and the structural variations among biosurfactants, which contribute to their unique physicochemical properties.

**Fig. 1.**
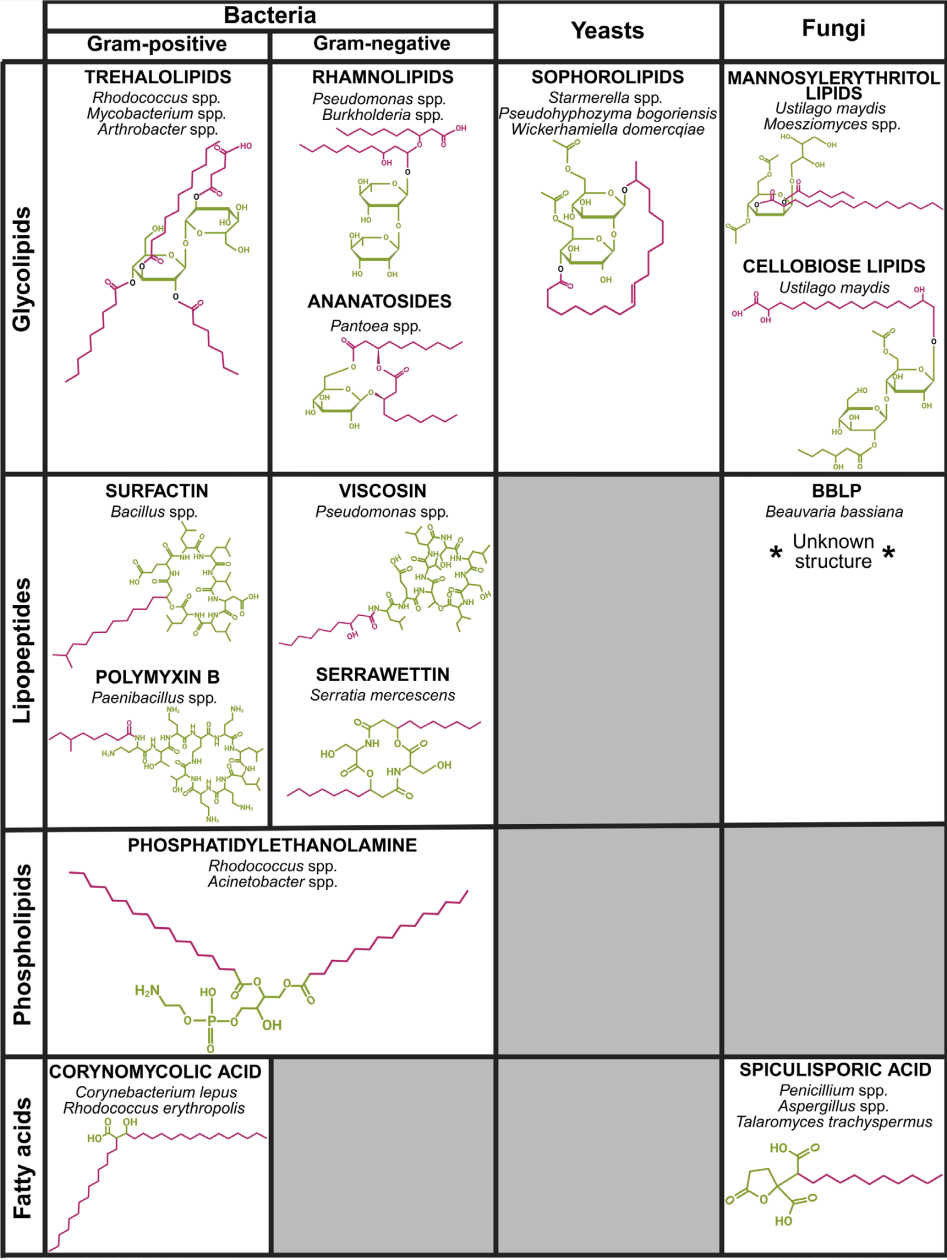
Classification of biosurfactants based on their chemical structure and microbial origin. This schematic table illustrates the diversity of biosurfactant types produced by various micro-organisms. The hydrophilic head is represented in green, and the hydrophobic tail is represented in purple. Representative examples from each subclass are shown: trehalolipid tetraester from *Rhodococcus erythropolis*; C_10_–C_10_ di-RL from *P. aeruginosa*; ananatoside A from *Pantoea ananatis*; diacetylated lactonic sophorolipid C_18 : 1_ from *S. bombicola*; mannosylerythritol lipid A from *Moesziomyces antarcticus*; cellobiose lipid B from *Ustilago maydis*; surfactin A from *B. subtilis*; polymyxin B1 from *Paenibacillus polymyxa*; viscosin from *Pseudomonas libanensis*; serrawettin W1 from *Serratia marcescens*; *Beauveria bassiana* LP (BBLP; structure not available); phosphatidylethanolamine from *Acinetobacter calcoaceticus*; corynomycolic acid from *Corynebacterium lepus*; spiculisporic acid from *Penicillium spiculisporum*. Figure created with BioRender.com

Biosurfactants constitute a heterogeneous family of molecules synthesized by various micro-organisms. They can be classified according to their chemical structure, which includes GLs, lipopeptides (LPs), phospholipids and fatty acids (Fig. 1). Among these classes, GLs are among the most well-studied due to their well-characterized properties. Structurally, they consist of a sugar moiety linked to a lipid tail. LPs, on the other hand, typically contain a nonribosomally synthesized peptide moiety attached to a fatty acid. Additionally, some micro-organisms can release modified phospholipids and fatty acids that exhibit tensioactive properties.

Furthermore, micro-organisms can produce multiple variants of a given biosurfactant, referred to as congeners, which differ by chemical modifications that alter their physicochemical properties. For instance, the yeast *S. bombicola* can synthesize over 100 sophorolipid congeners, varying in lipid tail length, degree of unsaturation, acetylation and the number of glucose units, depending on strains and culture conditions. These molecules can also exist in cyclic or linear forms. Similarly, *P. aeruginosa* produces RLs with lipid tails of different lengths, sometimes unsaturated, and with one or two rhamnose units. The diversity of congeners contributes to the structural and functional versatility of biosurfactants, potentially fulfilling distinct ecological roles within the natural environment of the producing micro-organisms. Indeed, *S. bombicola* produces both cyclic (lactonic) and linear (acidic) sophorolipids, which exhibit distinct properties. Lactonic sophorolipids tend to be more hydrophobic and display stronger antimicrobial activity than their acidic form.

Biosurfactants are synthesized by a variety of micro-organisms, including bacteria, yeasts and fungi. The most studied among them include the bacterial genera *Pseudomonas*, *Bacillus*, *Rhodococcus* and the yeast *S. bombicola*. These micro-organisms thrive in various ecological niches, including marine ecosystems, soils, plant rhizospheres, wastewater and extreme environments. Despite the remarkable diversity of biosurfactant-producing micro-organisms, the types of surface-active secondary metabolites they synthesize and their distinct ecological niches, common patterns can be observed in their biosynthesis, regulation and function.

This primer will primarily highlight the most prominent biosurfactant producers, namely *Pseudomonas* and *Bacillus*, and to a lesser extent *Starmerella*, due to space constraints. However, it is essential to recognize the extensive diversity of producers, as new ones are frequently identified.

## Shared functions of biosurfactants

Secondary metabolites are not involved in primary metabolism but rather serve accessory functions. Biosurfactants provide several benefits to the producing organisms. Their properties are a direct result of their amphiphilic nature, and the biological roles for the producing micro-organisms are generally found in microbe–microbe and microbe–host interactions, such as symbiosis, pathogenesis and competition. Here are some prominent functions of biosurfactants. The ecological context of their production matters and is key to understanding their contributions.

### Antimicrobial activities/antagonism

 Some micro-organisms produce antibiotics to compete with other competitors. Biosurfactants possess ‘membrane-active’ amphiphilic properties due to their hydrophilic head and hydrophobic tail, which allows them to disrupt the integrity of microbial cell membranes. Both GLs and LPs have been extensively investigated for their potential as antimicrobials. Especially, LPs exhibit growth-inhibitory activities against a broad range of micro-organisms, including bacteria, oomycetes and fungi. Iturins and fengycins are mostly known for their antifungal activities, while surfactins are broad-host-range antibacterials. Commercialized antibiotics such as polymyxin B and daptomycin are surface-active CLPs that penetrate and quickly disrupt the outer cell membrane or cell wall of susceptible bacteria by interacting with lipopolysaccharides or phospholipids. The mode of action may also involve the formation of micelles.

### Social surface motility

 Bacteria express various types of motilities. While flagella enable swimming in aqueous phases, they can also support swarming motility, which involves the rapid movement of a bacterial population across a surface. This is often observed as colonies forming intricate, dendritic, flowery patterns on semi-solid agar in laboratory settings. This type of motility typically requires the production of a surface-active agent that reduces viscosity and surface tension. A common feature of all bacterial swarmers is their ability to release sufficient levels of a surfactant to alter the environment around the colony. In natural conditions, swarming motility is thought to promote access to nutrients and water, for instance, by improving leaf wettability, thus facilitating surface colonization by phytopathogens such as *Pseudomonas syringae*.

### Biofilm development

 Bacterial communities adhere to surfaces and form biofilms, which are structured communities of adhered micro-organisms. Biosurfactants often play a crucial role in the formation*,* development, maintenance and dispersal of these biofilms by mediating the attachment and detachment of bacterial cells. Biosurfactants could contribute to this process through mechanisms such as cell surface modification and modulation of swarming motility. Additionally, the production of RLs plays a crucial role in maintaining open channels within the biofilm matrix. This helps facilitate the transport of nutrients, waste and metabolites throughout the biofilm.

### Virulence

 Through a combination of the above-mentioned properties, biosurfactants are considered virulence factors in the context of infections, despite their generally non-toxic and eco-friendly nature. Firstly, they typically display cytotoxic properties, as their amphiphilic nature promotes eukaryotic cell disruption. For instance, RLs were initially described as heat-stable haemolysins of *P. aeruginosa*. Similarly, several CLPs, such as tolaasin I, are important virulence factors produced by phytopathogenic *Pseudomonas* species; they contribute to disease by inducing membrane leakage through pore formation. Secondly, RLs have been shown to broadly modulate the host immune response, leading to tissue damage through various mechanisms such as neutrophil recruitment, stimulation of proinflammatory interleukin release and chemotaxis of polymorphonuclear leukocytes.

### Assimilation of hydrophobic substrates

 Finally, biosurfactants are also thought to enhance the uptake and biodegradation of poorly soluble substrates through emulsification and/or pseudosolubilization. They may also facilitate direct adherence to oil, hydrocarbon droplets or long-chain fatty acids by increasing cell surface hydrophobicity. However, it remains unclear whether microbial surfactant production has been evolutionarily selected to improve their access to hydrophobic substrates.

Overall, biosurfactants act as important mediators of interactions between the producing micro-organism and its environment and other organisms. However, the specific ecological role of their production often remains unclear. A first step towards clarifying their function is to understand how these molecules are synthesized and what biological components are involved in their production, an aspect presented in the following section.

## Common patterns in production

LPs constitute the largest class of biosurfactants. Primarily produced by soil and plant bacterial isolates, especially those belonging to the *Bacillus* and *Pseudomonas* lineages, these amphiphilic molecules are synthesized by large nonribosomal peptide synthetases (type I NRPS). These multimodule enzymatic complexes catalyse the formation of a peptide by linking amino acid residues that are attached to a linear or branched fatty acid with different lengths and degrees of oxidation. The polar peptidyl portion is either cyclized or not and can have a length ranging from 4 to 25 l- and d-amino acids, depending on the molecules produced. This results in an exceptional diversity of structures.

Among LPs, CLPs represent a structurally rich subclass, especially within the *Pseudomonas fluorescens* lineage. Although several distinct CLP groups have been identified, each containing multiple structurally homologous members, the viscosin and amphisin families are the most extensively represented. New CLPs are continuously identified, and they can usually be assigned to 1 of, at least, 14 previously described groups.

In the *P. fluorescens* lineage, LP producers are mostly found within the *P. fluorescens*, *Pseudomonas putida* and *P. syringae* phylogenetic groups, which include both plant-beneficial bacteria and phytopathogens. The biosynthesis of CLPs by *Pseudomonas* type I NRPS follows an ‘assembly line’ strategy. In this process, the peptide sequence is determined by the order of the modules acting sequentially, each responsible for the addition of one amino acid. Each elongation module usually contains three parts: a condensation domain, an adenylation domain and a thiolation domain. The fatty acid first attaches to an amino acid by a dedicated condensation domain in the initiation module. Subsequent amino acid additions are catalysed by a regular condensation domain, often with an additional epimerization function that converts some d-amino acids into the l-configuration. The transesterification domains of a pair of thioesterases carry out the final cyclization.

Most CLP-producing pseudomonads possess a single NRPS system. Therefore, a given species typically synthesizes one or more CLP congener(s) belonging to a particular structural family. Within each family, congeners display some variation in the saturation of the fatty acid moiety and/or in the amino acid composition and stereochemistry of a peptide chain of a defined length.

Beyond the *Pseudomonas* genus, LP production has also been well characterized in *Bacillus*, particularly through the study of surfactins synthesized by *B. subtilis* and closely related species such as *Bacillus amyloliquefaciens* and *Bacillus velezensis*. They are noncationic CLPs containing a macrolactone ring-shaped hexapeptide and a *β*-hydroxy fatty acid chain. Like many LPs, the isoforms of surfactins also vary according to the sequence and type of amino acids and the length of the lipidic portion. Usually, various surfactin congeners are coproduced as a mixture of several hexapeptide variants with different aliphatic chain lengths.

**Fig. 2.**
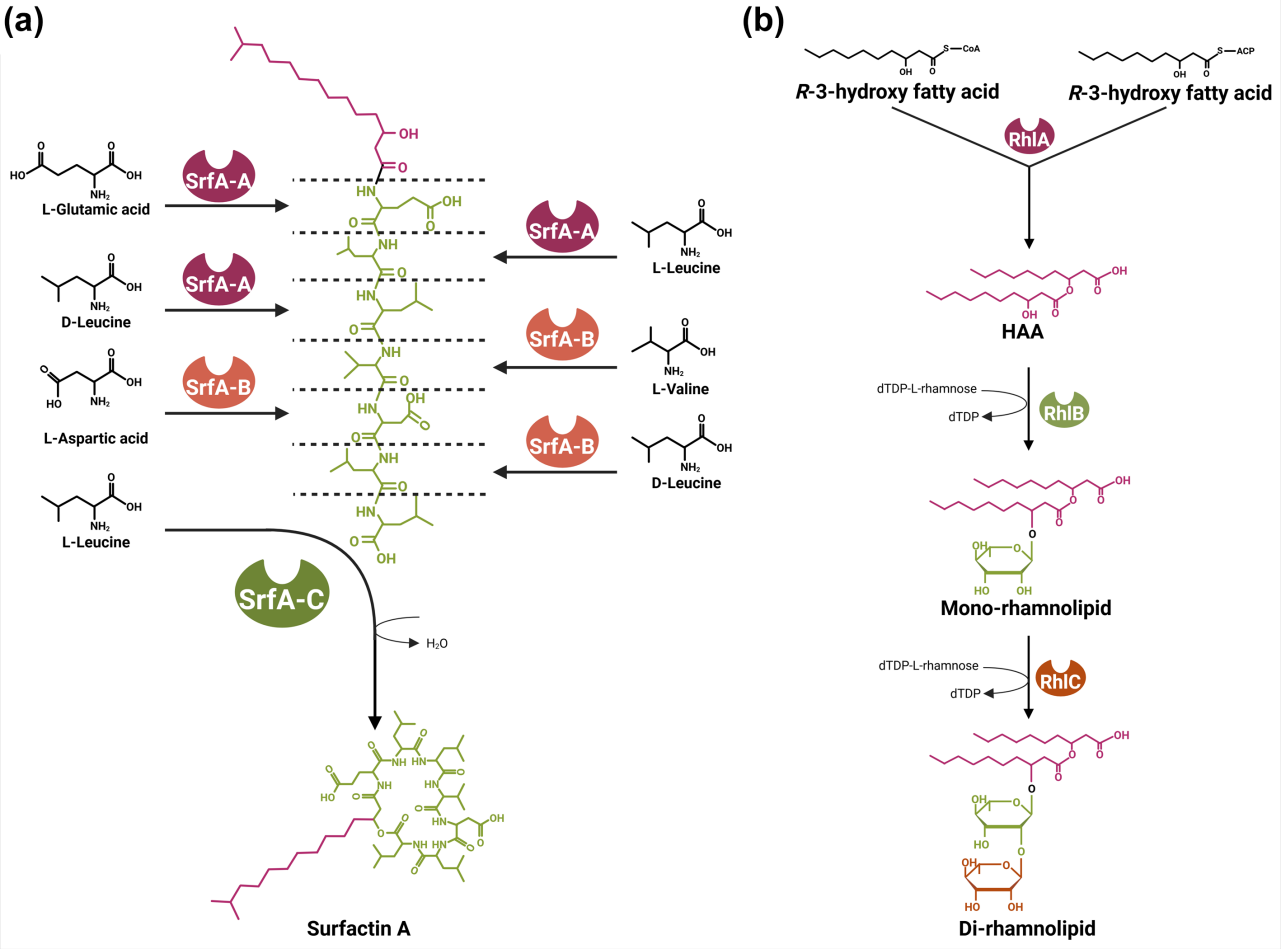
Schematic representation of the biosynthetic pathways of well-known biosurfactants. (**a**) Surfactin is synthesized by modular NRPS complexes in *B. subtilis*, which sequentially links amino acids and attaches a *β*-hydroxy fatty acid to form a CLP. (**b**) RL biosynthesis in *P. aeruginosa* involves the sequential action of RhlA, RhlB and RhlC enzymes, which respectively synthesize the lipid precursor (HAA), attach rhamnose units and form mono- and di-RLs. Figure created with BioRender.com.

The surfactin NRPS biosynthetic gene cluster in *B. subtilis* follows the canonical assembly line approach and consists of four biosynthetic subunits (SrfAA, SrfAB, SrfAC and SrfAD) (Fig. 2a). These proteins form seven catalytic modules, each corresponding to a specific amino acid in the final structure. Each module contains three key domains: an adenylation domain responsible for the selection and activation of the substrate, a small peptidyl carrier protein domain carrying the aminoacyl-adenylate substrate and a condensation domain that forms the peptide bonds between the intermediates. The final step involves the thioesterase domain of SrfAC, which catalyses the cyclization of the final leucine residue with a 3-hydroxy fatty acid, resulting in the release of the LP. In contrast to *Pseudomonas*, *B. subtilis* has separate epimerization domains that facilitate the conversion of certain incorporated residues to their d-isomer forms. Although the prototypical surfactin congener has the sequence (l-Glu)-(l-Leu)-(d-Leu)-(l-Val)-(l-Asp)-(d-Leu)-(l-Leu), the adenylation domains in modules 2, 4 and 7 display a promiscuous specificity, which contributes to a diversity of structures in the polar peptidyl portion.

Far fewer linear LPs have been identified, possibly because lactonization (cyclization) confers stability and bioactivity, which has diverted attention from them and limited characterization of their surfactant properties. Indeed, linear LPs display a reduced ability to lower surface tension compared to CLPs and are mostly known for their antimicrobial activities. The best-known linear LPs (e.g. cerexins, tridecaptins, corrugatins and syringafactins) have been isolated from cultures of *Bacillus* spp., *Paenibacillus* spp. and *Pseudomonas* spp. and are synthetized through an NRPS-type assembly line. The main distinction between cyclic and linear LPs lies in the function of the C-terminal thioesterase domain. In the biosynthesis of CLPs, this domain catalyses an intramolecular cyclization reaction that forms a ring structure upon releasing the peptide from the NRPS. For linear LPs, the termination domain primarily facilitates the release of the peptide chain as a linear molecule, without catalysing any intramolecular bond formation between the ends of the peptide.

In contrast with LPs, GLs are less diverse but accumulate in much higher quantities in cultures of producers. In general, the formation of glycolipidic biosurfactants involves the parallel biosynthesis of the polar moiety (sugar) and lipidic precursors. They are then linked together by dedicated enzymes, typically glycosyltransferases or acyltransferases, respectively, catalysing the formation of O-glycosidic or ester bonds.

RLs, originally identified in cultures of *P. aeruginosa*, and later found to be produced by several *Burkholderia* species, require three enzymes encoded by homologues of the *rhlA*, *rhlB* and *rhlC* biosynthetic genes. First, the 3-(3-hydroxyalkanoyloxy)alkanoate synthase RhlA dimerizes *R*-3-hydroxy fatty acids to form congeners of *R*-3-(*R*-3-hydroxyalkanoyloxy)alkanoic acids (HAAs). This lipidic precursor is then linked to a first rhamnose residue by the RhlB rhamnosyltransferase, followed by the addition of a second rhamnose by the RhlC rhamnosyltransferase (Fig. 2b). In *P. aeruginosa*, *rhlAB* and *rhlC* are carried on two distinct operons, while they are found on the same biosynthetic gene clusters in RL-producing *Burkholderia*.

Although homologues of these three genes are readily found in various bacterial species, only those that minimally encode *rhlA* and *rhlB* on the same operon synthesize GLs. It is noteworthy that diverse congeners are typically produced by a given species, containing various lengths of fatty acid side chains, or only one rhamnose residue. A similar strategy is used by the yeast *S. bombicola* to synthesize acidic sophorolipids, where a hydroxylated fatty acid is first produced by the action of the CYP52M1 cytochrome P450 monooxygenase. Then, the UgtA1 glycosyltransferase catalyses the formation of a glycosidic bond with a glucose residue, and UgtB1 adds a second glucose to form the disaccharide sophorose moiety. This process results in a wide variety of congeners, as these acidic sophorolipids can then undergo various levels of acetylation and lactonization.

Despite the remarkable structural and taxonomic diversity of biosurfactants, their biosynthesis follows conserved biosynthetic principles (Fig. 2). These shared features point to common regulatory mechanisms, which often involve population-level networks, a topic that will be discussed in the next section.

## Complex population-level regulatory networks

The regulation of biosurfactant biosynthetic genes often involves the interplay of multiple regulatory mechanisms, creating intricate control systems that respond to various internal and external cues. First, biosurfactant production is typically tightly linked to environmental conditions, ensuring its occurrence when most beneficial to the producing micro-organisms. Hence, nutrient availability, such as the presence of specific substrates and limitations in precise elements (carbon:nitrogen ratio, iron levels, etc.), is usually a key factor influencing the production of biosurfactants. Strikingly, it generally seems that cell density is an important factor controlling the expression of biosurfactant biosynthetic genes. Indeed, quorum sensing plays a significant role in coordinating biosurfactant production at the population level in several bacterial species. This regulation typically further integrates environmental and nutritional cues.

Quorum-sensing regulatory systems, which are mediated by cell-to-cell signalling, are exploited by most bacteria to regulate certain functions according to cell density. As presented above, the biological roles of amphiphiles, as mediators of interactions with the environment and other organisms, likely explain why production of these molecules has evolved to be regulated at the population level according to cell density. For a biosurfactant to effectively reduce surface tension, solubilize hydrophobic substrates or contribute to biofilm formation, a certain threshold concentration in the local environment is necessary. Quorum sensing ensures that production is increased only when there are enough cells present to collectively achieve an effective concentration of biosurfactant. Producing these compounds at low cell densities would be energetically costly and potentially futile. By linking biosurfactant biosynthesis to cell density, the entire community invests in production simultaneously, maximizing the overall impact and sharing the metabolic burden. This would also prevent the premature or wasteful synthesis of these compounds when the cell population is too sparse to benefit from them.

Quorum sensing mediated by acyl-homoserine lactone signals produced by LuxI-type synthases seems to regulate the transcription of biosynthetic genes in all RL-producing bacterial species, including *P. aeruginosa* and several *Burkholderia* species. The prototypical quorum-sensing regulation of biosurfactant production was originally elucidated 30 years ago in *P. aeruginosa*. In this bacterium, the synthase RhlI produces the diffusible signal butanoyl-homoserine lactone (C_4_-HSL), which acts as an activating ligand of the transcriptional regulator RhlR. The RhlR/C_4_-HSL complex then binds to a specific sequence in the *rhlAB* regulatory region to activate transcription. Therefore, the expression level of *rhlAB* depends on the concentration of C_4_-HSL. The second rhamnosyltransferase, encoded by *rhlC*, is coordinately regulated with *rhlAB* by the same quorum-sensing regulatory system.

Due to the large diversity in fluorescent *Pseudomonas* species, which produce a plethora of different LPs, no model system has been established. As a result, most knowledge on the regulation of LP biosynthesis in the *P. fluorescens* lineage remains limited. Only a few CLPs in this lineage, such as putisolvins by *P. putida* or viscosins by *P. fluorescens*, are regulated by a typical LuxR/acyl-homoserine-lactone quorum-sensing system like in *P. aeruginosa*. Interestingly, while the NRPS biosynthetic genes in the *P. fluorescens* lineage are usually controlled by LuxR-type regulators, most species do not seem to produce a cognate acyl-homoserine lactone signal. Instead, they regulate their LuxR homologues post-transcriptionally through complex Gac/Rsm regulatory cascades. The GacS/GacA two-component systems respond to still unknown environmental signals and control the availability of post-transcriptional repressor proteins, such as RsmA and RsmE.

In *Bacillus* species, the complex regulation of surfactin production has also been extensively investigated. While the expression of the *srfAA-AD* operon is regulated by several regulatory elements, the main control system relies on ComX pheromone peptide‐mediated quorum sensing. This is a typical quorum-sensing regulatory system found in *Bacillota* (*Firmicutes*) bacteria. The ComP-ComA two-component system is the primary regulatory system governing transcription of the *srfAA-AD* operon. The biosynthesis of surfactin begins with the autoinducer ComX, a peptide pheromone that is constantly released during cell growth. ComX is produced as a precursor, which undergoes post-translational modification and is then secreted. The sensor kinase ComP binds the extracellular ComX signal when it reaches a certain threshold concentration and subsequently phosphorylates the ComA response regulator. The phosphorylated ComA then activates the transcription of the *srfAA-AD* operon by binding to the P*_srf_* promoter, inducing surfactin production.

Altogether, while quorum sensing and multitiered regulatory mechanisms have been well described in bacteria, still very little is known about the regulation of biosurfactant biosynthesis in eukaryotic producers such as *Starmerella* spp. and *Moesziomyces* spp.

## Conclusion

In summary, microbial surfactants play essential roles in microbial physiology and ecology, particularly influencing microbe–microbe interactions, environmental colonization and the regulation of microbial communities. Understanding these properties is fundamental for studying microbiomes, as biosurfactants enhance the survival and cooperation of diverse microbial populations in dynamic and often extreme environments. They are also crucial for microbial persistence on surfaces, both in industrial contexts and in medical settings, where biofilm-related infections pose significant challenges.

From a biotechnological perspective, biosurfactants are emerging as the most promising sustainable alternative to synthetic surfactants. While synthetic surfactants are commonly used in products such as detergents, soaps and various industrial processes, most are derived from petroleum, are non-biodegradable, are often toxic and generate harmful waste, leading to considerable environmental risks. In contrast, biosurfactants exhibit eco-friendly properties and a broad range of applications, making them an appealing alternative with numerous industrial advantages. Therefore, these molecules hold strong potential for use in food products as additives and preservatives, in cosmetics and pharmaceuticals as emulsifiers and in environmental applications such as oil recovery and bioremediation.

Current research on biosurfactants focuses on two key areas: commercial and fundamental research, with the former being the predominant field of study. In commercial research, efforts are directed at exploring the potential of biosurfactants in various industrial applications while optimizing their production processes and downstream uses. On the other hand, fundamental research continues to explore the ecological roles and functions of biosurfactants within natural microbial ecosystems, including their associations with human and animal infections.

Ultimately, the investigation of microbial surfactants represents a convergence of ecological, biotechnological and medical research, providing considerable opportunities for innovation and improving our understanding of microbial life.

## Further reading

Abdel-Mawgoud AM, Lépine F, Déziel E. Rhamnolipids: diversity of structures, microbial origins and roles. *Appl Microbiol Biotechnol* 2010;86:1323–1336. 10.1007/s00253-010-2498-2

Cesa-Luna C, Geudens N, Girard L, De Roo V, Maklad HR, *et al*. Charting the lipopeptidome of nonpathogenic *Pseudomonas*. *mSystems* 2023;8:e0098822. 10.1128/msystems.00988-22

Cochrane SA, Vederas JC. Lipopeptides from *Bacillus* and *Paenibacillus* spp.: A gold mine of antibiotic candidates. *Med Res Rev* 2016;36:4–31. 10.1002/med.21321

Dierickx S, Castelein M, Remmery J, De Clercq V, Lodens S, *et al*. From bumblebee to bioeconomy: recent developments and perspectives for sophorolipid biosynthesis. *Biotechnol Adv* 2022;54:107788. 10.1016/j.biotechadv.2021.107788

Marchant R, Banat IM. Microbial biosurfactants: challenges and opportunities for future exploitation. *Trends Biotechnol* 2012;30:558–565. 10.1016/j.tibtech.2012.07.003

Raaijmakers JM, De Bruijn I, Nybroe O, Ongena M. Natural functions of lipopeptides from *Bacillus* and *Pseudomonas*: more than surfactants and antibiotics. *FEMS Microbiol Rev* 2010;34:1037–1062. 10.1111/j.1574-6976.2010.00221.x

Sarubbo LA, Silva M da GC, Durval IJB, Bezerra KGO, Ribeiro BG, *et al*. Biosurfactants: production, properties, applications, trends, and general perspectives. *Biochem Eng J* 2022;181:108377. 10.1016/j.bej.2022.108377

Théatre A, Cano-Prieto C, Bartolini M, Laurin Y, Deleu M, *et al*. The surfactin-like lipopeptides from *Bacillus* spp.: natural biodiversity and synthetic biology for a broader application range. *Front Bioeng Biotechnol* 2021;9:623701. 10.3389/fbioe.2021.623701

Twigg MS, Baccile N, Banat IM, Déziel E, Marchant R, *et al*. Microbial biosurfactant research: time to improve the rigour in the reporting of synthesis, functional characterization and process development. *Microb Biotechnol* 2021;14:147–170. 10.1111/1751-7915.13704

Zhou L, Höfte M, Hennessy RC. Does regulation hold the key to optimizing lipopeptide production in *Pseudomonas* for biotechnology? *Front Bioeng Biotechnol* 2024;12:1363183. 10.3389/fbioe.2024.1363183

Zibek S, Soberón-Chávez G. Overview on glycosylated lipids produced by bacteria and fungi: Rhamno-, sophoro-, mannosylerythritol and cellobiose lipids. *Adv Biochem Eng Biotechnol* 2022;181:73–122. 10.1007/10_2021_200

## Abbreviations

C_4_-HSL: butanoyl-homoserine lactone
CLP: cyclic lipopeptide
GL: glycolipid
HAA: *R*-3-(*R*-3-hydroxyalkanoyloxy)alkanoic acid
LP: lipopeptide
NRPS: nonribosomal peptide synthetase
RL: rhamnolipid

